# Geographical distribution and the impact of socio-environmental indicators on incidence of Mpox in Ontario, Canada

**DOI:** 10.1371/journal.pone.0306681

**Published:** 2025-03-11

**Authors:** Chigozie Louisa J. Ugwu, Ali Asgary, Jianhong Wu, Jude Dzevela Kong, Nicola Luigi Bragazzi, James Orbinski, Woldegebriel Assefa Woldegerima

**Affiliations:** 1 Laboratory for Industrial and Applied Mathematics (LIAM), Department of Mathematics and Statistics, York University, Toronto, Ontario, Canada; 2 The Advanced Disaster, Emergency and Rapid Response Program, York University, Toronto, Canada; 3 Africa-Canada Artificial Intelligence and Data Innovation Consortium (ACADIC), Toronto, Canada; 4 Global South Artificial Intelligence for Pandemic and Epidemic Preparedness and Response Network (AI4PEP),; 5 Artificial Intelligence & Mathematical Modeling Lab (AIMM Lab), Dalla Lana School of Public Health, University of Toronto, Toronto, Canada; 6 Department of Health Sciences (DISSAL), Postgraduate School of Public Health, University of Genoa, Genoa, Italy; 7 United Nations Educational, Scientific and Cultural Organization (UNESCO) Chair, Health Anthropology Biosphere and Healing Systems, University of Genoa, Genoa, Italy; 8 The Dahdaleh Institute for Global Health Research, York University, Toronto, Canada; Prince Sattam bin Abdulaziz University, SAUDI ARABIA

## Abstract

**Background:**

Ontario, being one of Canada’s largest provinces, has been central to the high incidence of human Mpox. Research is scarce on how socio-environmental factors influence Mpox incidences. This study seeks to explore potential geographical correlations and the relationship between indicators of social marginalization and Mpox incidence rate in Ontario.

**Methodology:**

We used surveillance data on confirmed human Mpox cases from May 1, 2022, to March 31, 2024, extracted from the Public Health Ontario website for this study. Spatial autocorrelation of Mpox incidence was investigated using spatial methods including Moran’s Index, Getis–Ord Gi*statistic, and spatial Poisson scan statistic. Following this, we adopted a generalized Poisson regression (GPR) model to estimate the incidence rate ratios (IRRs) based on the association between Ontario PHU-level marginalization and Mpox incidence, while adjusting for age and sex. The goodness-of-fit of the models was assessed using the Log Likelihood (LL), Akaike Information Criterion (AIC), Akaike’s Information Criterion corrected (AICc), and the Bayesian Information Criterion (BIC).

**Results:**

Our analysis revealed significant localized spatial heterogeneity in Mpox incidence across Ontario. Statistically significant local clusters of Mpox cases were identified in Toronto (RR=11.34,LL=511.97 ), Ottawa (RR=3.21,LL=19.88), and a secondary cluster, overlapping Hamilton PHU with nine local districts (RR=2.07,LL=9.64), all with p−value<0.05. The incidence rate of Mpox was statistically significantly associated with a higher proportion of ethnic concentration (racialized groups, migrants, or visible minorities) $\left(IRR=9.478;95\%CI=5.062-17.747\right)$, gender $\left(IRR=5.150;95\%CI=1.159-8.324\right)$ and higher residential instability $\left(IRR=14.112;95\%CI=6.596-30.189\right)$.

**Conclusion:**

We identified major Mpox hotspots in Toronto. According to our model results, the high incidence rate may be influenced by the greater population of internal migrants and younger individuals. Based on these insights, we recommend targeted interventions in the high-risk neighborhoods. Efforts to improve Mpox diagnosis and promote health equity among socioeconomically vulnerable populations, including racial and ethnic minorities, should be implemented.

## Introduction

In July 2022, the World Health Organization (WHO) declared the Mpox disease to be a pandemic due to its sporadic spread to non-endemic regions [1,2]. The global public health, social security, and the economy have been challenged by the Mpox outbreak in non-endemic areas, including Canada [1]. In September 2023, the virus had infected over 90,000 people worldwide, and 117 countries had reported cases of deaths associated with the Mpox. Recent epidemiological studies from non-endemic countries have highlighted large differences in the incidence of Mpox in the context of geographical locations, population, and socio-environmental factors [3–6].

In Canada, the Mpox burden is unevenly distributed geographically across provinces and within provinces, while the highest number of cases were witnessed in Ontario [2,7]. As of September 31, 2023, there were a total of 1,515 human Mpox cases in Canada and 722 human Mpox cases in Ontario alone, with no fatalities [1,8].

Although vaccination and public health measures have been successful in reducing the spread of Mpox in Canada, the province of Ontario still observed an increase in Mpox occurrence from mid-January to the end of March 2024 with a total of 32 confirmed human Mpox cases, despite the decline across the country [9,10]. The new occurrence of Mpox cases suggest a possible disproportionate burden of confirmed human Mpox cases in Ontario in relation to socio-environmental and behavioral determinants [11].

In Canada, there is limited research on the role of social and demographic factors that may influence both the incidence and within province spread of Mpox [2,9]. This is an important area of study considering prior work conducted in Ontario in the context of similar diseases like Covid-19, where researchers found that people that are marginalized had increased risk of COVID-19 morbidity and mortality as compared to the general population in Ontario [12–14]. This may demonstrate possible lack of health service equity [15].

It is epidemiologically recognized that socioeconomic status, marginalization, and inequity are significant risk factors for morbidity and mortality from various diseases [16,17]. Research has shown that a person’s socio-economic position, which includes factors like education, occupation, and income, can have a significant impact on their overall health [6]. Typically, individuals with a lower socio-economic status are more susceptible to health issues and disease infection, hence, contributing to health inequities that impact individuals across all levels of society [1,18].

In Ontario, the largest province in Canada, and most demographically diverse province in the country, vulnerability to disease infection could be influenced by the level of marginalization of the PHUs or neighborhood in which individuals live [19,20]. Although there is prior research on health equity and other diseases such as COVID-19 in the context of marginalization, there has not been any study on the role of social factors in the risk of contracting Mpox in Ontario.

Much work has been done in other non-endemic countries like the USA, European countries and in Brazil, with less literature in Canada [21,22]. Research evidence in other countries has shown that marginalized groups of individuals are more likely to be vulnerable to Mpox infection [6,23,24]. Apart from sexually diverse groups, ethnic minorities and socioeconomically disadvantaged (material deprived) individuals may have disproportionately suffered socio-determinants of health, with disproportionate resource distribution, societal exclusion, and different life experiences having well-documented implications for health status and well-being [19]. Understanding the epidemiological context, geographical distribution, and influence of socio-environmental indicators in Mpox incidence at PHU level in Ontario can be an effective strategy for planning public health policies and targeting priority areas.

Given the availability of the 2021 Ontario marginalized index data [25,26], this study aims to examine the geographical distribution and the role of PHU-level marginalization indicators on the incidence of Mpox cases in Ontario, while adjusting for age and gender. We hypothesize that (1) Mpox infection rates are randomly distributed among the PHUs in Ontario (values of the features, i.e., Mpox incidence rates throughout Ontario are spatially uncorrelated) and (2) PHUs with higher population density and greater levels of marginalization are likely to experience an increased incidence of Mpox. This study could guide resource allocation, including testing, vaccination strategies, and other disease control interventions, to Public Health Units (PHUs) at higher risk of Mpox.

## Materials and methods

### Study area and data

**Fig 1:**
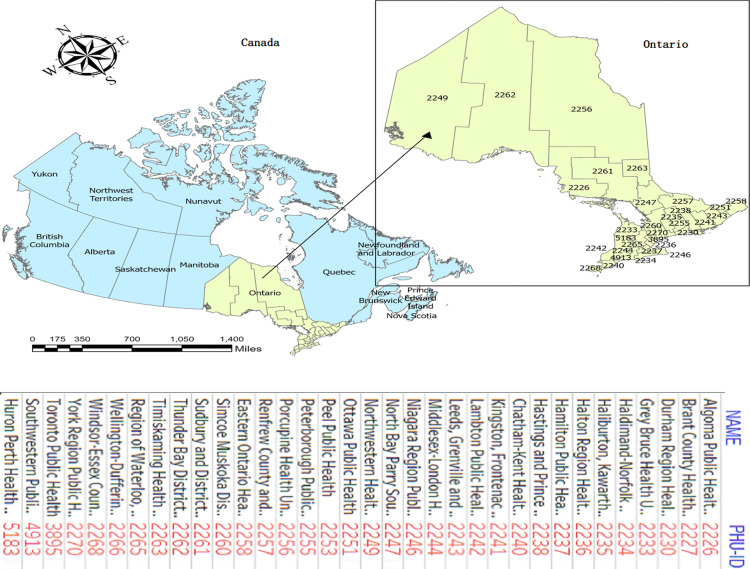
Geographic location of the study area. Ontario is in east-central Canada and the country’s second-largest province by land area in km^2^. Shape file source: Statistics Canada 2021, URL: https://www12.statcan.gc.ca/census-recensement/2021/geo/sip-pis/boundary-limites/index2021-eng.cfm?year=21. Map produced by authors using ArcGIS Pro version 3.0.3.

The study region, as shown in **Fig 1**, is the province of Ontario in Canada, which is partitioned into 34 units called Ontario Public Health Units (PHUs). Ontario is a highly populated province in the east-central part of Canada. Ontario has an estimated population of 14.2 million, a land area of 892,411.8 $km^{2}$ with a population density of 16 inhabitants/ $km^{2}$ [27]. Approximately 34% of the population are visible minorities, and about 44% are immigrants born outside Canada [28].

The PHUs, which were formed by grouping several urban and rural municipalities, are the smallest geographical units considered for this study [28]. Algoma (2226), Northwestern (2249), Thunder Bay District (2262), Sudbury and District (2261), and the Timiskaming PHUs are situated in Northern Ontario, while the remaining 29 PHUs make up Central, Toronto, Southern and Eastern Ontario Health Unit Regions [28].

### Mpox epidemiological data

We obtained data on confirmed human Mpox cases in Ontario from May 1, 2022, to March 31, 2024, from the Integrated Public Health Information System (iPHIS) of Public Health Ontario (PHO), which records cases of reportable Mpox disease across Ontario and publish as surveillance report [9,10]. Between May 1, 2022, and July 2023, Ontario reported a total of 722 confirmed Mpox cases, with no deaths associated with the disease [11]. Notably, there was a resurgence in Mpox cases beginning in mid-January 2024, with 32 confirmed cases reported by the end of March 2024, slightly fewer than the 33 cases recorded throughout 2023 [9]. Overall, our study included a total of 758 Mpox cases across Ontario’s PHUs. We utilized the 34 PHUs $\left(n=34\right),$ as the spatial unit of analysis. All Mpox cases were geographically linked to the corresponding PHU using boundary polygons in ArcGIS Pro [29]. These PHU-level polygons (geographical shapefiles) were sourced from the publicly accessible Statistics Canada Geodatabase [28,30].

### Population and socio-demographic data

Based on existing studies and data availability, our explanatory variables included a variety of socioeconomic, and demographic factors initially retrieved at PHU level from the 2021 Statistics Canada population census data [30,31]. Apart from the population density, age distribution and gender, these variables included: (1) percentage of the population aged 15–64 years with a lower level of education (not having a university certificate, diploma or a bachelor degree), (2) prevalence of low income (living in a low-income household based on based on the Low-income measure, after tax (LIM-AT), table representing the poverty line), (3) unemployment rate (population over 15 years and unemployed), (4) percentage of immigrants (individuals who were born outside of Canada), and (5) average household size.

However, we found that the listed variables from 1 to 5 are subset variables used to construct the 2021 Ontario Marginalized index (ON-Marg) factors, a widely used index that encompass various factors of socioeconomic and marginalization status at Ontario PHU level [25,26]. The 2021 ON-Marg Index was created through principal component analysis from the 2021 Canadian census data, jointly by researchers at MAP Centre for Urban Health Solutions at St. Michael’s Hospital and Public Health Ontario [25].

These are four dimensions (subdomains) of marginalization as measured by the 2021 ON-Marg for each PHU in Ontario and have been used extensively across Ontario for research purposes on health and disease disparities, advocacy work, population health assessment and surveillance, and public health program planning and resource allocation [25,32]. Therefore, as described in **Table 1**, this study extracted the 2021 ON-Marg index [26], for each PHU in Ontario, derived using principal component factor analysis of 42 indicators from the 2021 Census of Population [33,34]. Additionally, the four-dimensional ON-Marg factors have been demonstrated to be linked to many health outcomes and preferred for use in this research to overcome the issue of multicollinearity, and to demonstrate its potential to measure health inequalities in the context of Mpox incidences in Ontario.

**Table 1:** Explanatory variables.

Variable	Description
**Material resources or deprivation**	The ON-Marg dimension of material deprivation is closely **connected to poverty** (**low socio-economic status**) and analytically factored from the 2021 population census data using these indicators: (1) Proportion of the population aged 15 years ^+^ without a high-school diploma (low level of education). (2) Proportion of families who are lone parent families. (3) Proportion of the population aged 15 years^+^ who are unemployed. (4) proportion of the population considered low-income households (prevalence of low-income), and (5) Proportion of households living in dwellings that need major repair.
**Population density**	Percentage of people residing in each PHU per km/square (measurement of population per unit land area for each PHU).
**Household dwelling (residential instability)**	Composite measure of: (1) Proportion of population living alone. (2) Average number of individuals per household (dwelling) (3) Proportion of the population aged 15^+^ (4) Proprtion of population who are single, divorced, or widowed.
**Racialized and newcomer populations (ethnic concentration or visible minority)**	Composite measure of: (1) Proportion of population whoa are recent immigrants (within 5 years), and (2) Proportion of the population who self-identified as a visible minority (as defined by Statistics Canada), they are and/or non-white, non-Indigenous populations.
**Age and labour force (dependency)**	Composite measure of: (1)Proportion of the population who are 65 + (2) Proportion of population not participating in labour force (aged 15+) (3) Dependency ratio (total population aged 0 to 14 and 65 + over the total population aged 15 to 65 years.
**Gender**	The proportion of males aged 15 + of the total population of males and females residing in each PHU.
**Percentage age 0-14 year**	Age groups of the population at each PHU from 0-14 years
**Percentage age 15-64 year**	Age groups of the population at each PHU from 15-64 years
**Percentage age 65year and over**	Age groups of the population at each PHU from 64 years and over.
**Average household size**	The average number of persons per household.
**Married or common law partner**	Proportion of married or common law partners who set up their household together in one dwelling.
**For each dimension, scores were treated as a continuous variable from lower to higher level of marginalization**	

These dimensional factors (**Table 1**) included: material resources (previously called ‘material deprivation’), households and dwellings (previously called ‘residential instability’), age and labour force (previously called ‘dependency’) and racialized and newcomer populations (previously called ‘ethnic concentration’) or Percentage of visible minorities, have been associated with many health outcomes [25,26].

According to the Employment Equity Act*,* visible minorities are “persons, other than Aboriginal peoples, who are non-Caucasian in race or non-white in colour”. We also adjusted for demographic factors such as gender, and population density, and age distributions by PHU extracted from the 2021 Canadian census [31]. Population density was measured as the number of persons per square kilometer, and it was found to be highly skewed to the right and therefore, a log-transformation was applied.

Datasets were linked to the geographic boundary files, sourced from Statistics Canada and Ontario Open Data [31], using the PHU-ID in Arc GIS Pro. [29].

### Ethical considerations

The current study utilized publicly available Mpox data; therefore, ethical approval is not required. The data is available online at: https://www.publichealthontario.ca/

### Measurement of a dependent variable

The Mpox incident rate per 100,000 population for the years 2022-2023 was used as the dependent variable. To calculate this rate, we employed the 2021 census population data to ascertain the population at risk of Mpox for each PHU in Ontario, using the formula:


$${\left(MIR/100K\right)}_{PHUs\left(x\right)}=\left(\frac{TotalnumberofMpoxconfirmedcases_{PHUs\left(x\right)}}{TotalpopulationofeachPHUs_{\left(x\right)}}\right)\times 100,000$$

Subsequently, the Mpox incidence rate per 100 000 population for each PHU and the explanatory variables were linked to the PHU polygon in the ArcGIS Pro version 3.0.36056 for further analysis [35].

## Analytical methods

### Global spatial autocorrelation analysis

This study first applied spatial autocorrelation to determine whether Mpox cases are correlated geographically. Spatial autocorrelation, as determined by Global Moran’s Index [36], acts as the indicator for analyzing Mpox geographical distribution throughout Ontario. Spatial autocorrelation is closely related to Tobler’s first law of geography, which states that “everything is connected to everything else, but objects at proximity are more strongly interconnected than those farther apart.” [37]. Global Moran’s Index statistics equal to zero (0) shows the absence of spatial correlation, indicating a random distribution of Mpox, and no clustered PHUs across Ontario. A Global Moran’s Index >0, indicates the presence of positive spatial autocorrelation, and when the value approaches +1, it signifies a strong positive spatial autocorrelation, indicating that the PHUs are clustered.

In this study, we evaluated the spatial autocorrelation of the Mpox incidence rate throughout Ontario using the global Moran’s I, and the interpretation of the result was considered in the context of its null hypothesis of spatial randomness [36]. The null hypothesis specifies that the Mpox incidence are randomly distributed among the PHUs within Ontario. A p−value resulting from the Global Moran’s I test below the 5% significance level indicates the presence of spatial autocorrelation of Mpox among the PHUs across Ontario. Let, $x_{i}$ and $x_{j}$ denote observed Mpox cases at PHUs i and j,i,j=1,…,n=34. Then the global Moran’s I statistic [36], is defined as:


$$I=\frac{N\sum_{i=1}^{n}\sum_{j=1}^{n}w_{i,j}\left(x_{i}-\bar{y}\right)\left(x_{j}-\bar{X}\right)}{\left(\sum_{i=1}^{n}\sum_{j=1}^{n}w_{i,j}\right)\sum_{i=1}^{n}\left(x_{i}-\bar{X}\right)}i\neq j,$$ (1)

where *N* is the total number of Mpox cases, *n* is number of PHUs, $W_{i,j}$ denote the spatial weights between PHU i and PHU j;$\bar{X}$ represents the mean value of Mpox cases across the entire PHU and it is given by $\bar{X}=\frac{\sum_{i}x_{i}}{n}$, $x_{i}$ and $x_{j}$ denote the number of Mpox cases in PHU i and *j*, respectively,  .  The corresponding values of z− score and p−value of Moran’s I statistic are used to reject or accept the null hypothesis of spatial randomness of Mpox distribution across the entire PHUs in Ontario. For this study, we employed a fixed distance band spatial matrix for the autocorrelation (Global Moran’s I) analysis.

### Local indicators of spatial autocorrelation

#### Local Moran’s I statistic.

The major limitation of Global Moran’s I statistic is that it only generates a single summary statistic for the entire study area, meaning that it only measures spatial autocorrelation (clustering) at the global scale [38]. Global Moran’s I does not detect where local clusters or spatial outliers are located [39]. Even if global autocorrelation is not statistically significant, testing for local clusters ensures that localized Mpox patterns within specific PHUs are detected. Therefore, we applied the local Moran’s I statistic (LISA) introduced by [39], to investigate the local level of spatial clustering of PHUs with a high and low incidence of Mpox [38]. The computation of local Moran’s I assesses the local version of Global Moran’s I for each PHU, computes scores that reveal the underlying significant spatial clustering and local spatial outliers within the data at each PHU to determine variation in spatial autocorrelation over Ontario. Its significance is evaluated in five categories namely: High-High, High-Low, Low-High, Low-Low, and non-significant Mpox incidence rates [40]. LISA effectively pinpoints PHUs where Mpox incidences are significantly notable, offering insights into potential underlying mechanisms [39]. It calculates a local Moran’s I value, a z-score, a pseudo p-value, and a code representing the cluster type for each statistically significant PHU. The z-scores and pseudo p-values indicate the statistical significance of the computed Local Moran’s I values [39], at p−value<0.01.

Let $x_{i}$ be the ith Mpox observation at the ith PHU, then, the local Moran’s I statistic [41] of spatial association is calculated using the following formula:


$$I_{i}=\frac{x_{i}-\bar{X}}{S_{i}^{2}}\overset{n}{\underset{i=1,i\neq j}{\sum }}w_{i,j}\left(x_{i}-\bar{X}\right)$$ (2)

where $x_{i}$ is the Mpox incidence rate in the ith PHU; $w_{i,j}$ is the spatial weight matrix that defines spatial interaction between PHU *i* and j;*n* is the total number of PHUs, $\bar{X}$ is the mean and $S_{i}^{2}=\frac{\sum_{i=1,i\neq j}^{n}{\left(\left(x_{i}-\bar{X}\right)\right)}^{2}}{n-1}$ is the deviation of neighbouring PHUs [39]. This study employed the K-nearest neighbors’ approach to establish a spatial weight matrix, wherein the spatial autocorrelation relationship was defined with k=8 neighboring method, which produced similar but more reliable results when compared to other methods [41]. We used ArcGIS Pro (ESRI, Redlands, CA, USA) [35], LISA analysis. The statistical significance level was set at 0.05, and we used 9999 permutations in the simulation to assess the sensitivity of our results [41].

#### Getis-Ord local Gi * statistic.

As alternative to local Moran’s I inferential statistic, local Getis-Ord Gi * statistic was applied to statistically identify significant hot spots (areas with values significantly higher than the average) and cold spots (areas with significantly lower values) [42]. While PHU with a high Mpox count is noteworthy, it may not necessarily be a statistically significant hotspot. Unlike the local Moran’s I, the Getis-Ord Gi * statistic value computed in each PHU is expressed as z-score value, which allows a direct interpretation for statistical significant of the PHU [42]. The Getis- Ord Gi * statistic is computed using the following formula:


$$G_{i}^{*}=\frac{\sum_{j=1}^{n}w_{i,jx_{i}}-\bar{X}\sum_{j=1}^{n}w_{i,jx_{i}}}{\sqrt[{S}]{\frac{\left[n\sum_{j=1}^{n}w_{i,j}^{2}-{\left(\sum_{j=1}^{n}w_{i,j}\right)}^{2}\right]}{n-1}}}$$ (3)

where $x_{i}$ is the Mpox count at ith PHU, $w_{i,j}$ is the spatial weight between ith and jth PHU, *n* denote the number of PHUs, $\bar{X}$ is the mean and *s* is the standard deviation. We used ArcGIS Pro (ESRI, Redlands, CA, USA) [35], the Getis-Ord Gi * statistic, setting the number of permutation tests at 9999 for sensitivity analysis and a 0.05 level of significance. The K-nearest neighbors approach was used to conceptualize the spatial relationships [41].

#### Spatial Poisson scan statistics.

A key limitation of LISA and Getis-Ord hot spot analysis is their reliance on predefined spatial scales, which may limit their ability to detect Mpox clusters of varying sizes and shapes. To address this, we applied the Purely Spatial Poisson Scan Statistic using a discrete Poisson probability model [43], enabling a more flexible and comprehensive assessment of Mpox incidence across Ontario, capturing broader local spatial patterns [43]. This analysis aimed to identify statistically significant spatial clusters of Mpox, both high and low, for comparison with Mpox hotspots identified by the Getis-Ord Gi * statistic [44]. Unlike the LISA and Getis-Ord Gi * statistics, the retrospective purely spatial scan Poisson model detects most likely high clusters accurately because it fits the assumption that the number of Mpox cases in each PHU was Poisson distributed according to a known underlying population at the risk of Mpox.

The analysis employed a circular moving window centered on each PHU, with a maximum spatial cluster size based on the population at risk of Mpox. The window moved until reaching the maximum population at risk, with the radius of each circle continuously increasing to a maximum radius. The maximum likelihood function was used to maximize the circular window size, identifying the primary cluster as the circular window with the highest likelihood ratio [44]. Additional non-overlapping clusters in circular windows with high likelihood ratios were identified as secondary clusters. Each circular window was tested using a Monte Carlo simulation to assess the null hypothesis of spatial randomness. SaTScan version 10.1.3 was used for this analysis, with a maximum window size of 50% of the population at risk [45]. To ensure statistical power, 999 Monte Carlo replications were performed, considering only clusters at a 99% confidence interval as statistically significant.

### Non-spatial statistical analysis

In this study, we conducted a non-spatial statistical analysis of Mpox count incidence data obtained across the 34 PHUs in Ontario due to lack of significant global spatial autocorrelation. The standard Poisson regression (PR) model was applied as the initial analytical approach. This model is suitable for the count data, particularly when our response variables represents the Mpox cases occurring within a fixed observation period [46]. The standard PR model assumes equal mean and variance of the response variable, making it suitable only when the data do not exhibit overdispersion [47]. Let $Y_{i}$ be the count of Mpox in the *i* th PHU, i=1,2,…,34. If $Y_{i}\sim$ a Poisson distribution, the probability mass function (PMF) is expressed as:


$$f\left(Y_{i}=y_{i};\mu_{i}\right)=\frac{e^{-\mu_{i}}\mu_{i}^{y_{i}}}{y_{i}!},y_{i}=0,1\ldots .$$ (4)

where $Y_{i}$, is the count of Mpox cases in the *i* th PHU, $\mu_{i}$ is the mean or expected number of Mpox cases in the *i* th PHU, *e* is the Euler’s number (approximately 2.718), and $y_{i}$ is the observed count of Mpox cases. The Poisson model assumes that the mean equals the variance, $E\left(Y_{i}\right)=Var\left(Y_{i}\right)=\mu_{i}.$ To integrate the covariates $x_{i}$ into Equation (4) while ensuring non-negativity, the mean is presumed to be multiplicative, and we model the mean $\mu_{i}$ as a function of the covariates via the log-link function such that:


$$Y_{i}\sim \text{Poiss}\left(\mu_{i}\right),\log\left(\mu_{i}\right)=exp\left(x_{i}^{T}\beta \right)$$ (5)

where $\mu_{i}$ is the expected number of Mpox cases for the *i* th PHU, $E\left(Y_{i}\right)=Var\left(Y_{i}\right)=\mu_{i}=\text{exp}\left(x_{i}^{T}\beta \right)$, $x_{i}$ is the vector of covariates for the *i* th PHU (socio-environmental variables), and *β* is the vector of unknown regression coefficients.

To ensure robustness of the regression parameters *β*, we utilize the Maximum Likelihood Estimation (MLE) to estimate the coefficients of the model. The likelihood function for distribution is given by:


$$L(\beta |y_{i})=\overset{n}{\underset{i=1}{\prod }}\frac{e^{-\mu_{i}}\mu_{i}^{y_{i}}}{y_{i}!}$$ (6)

Taking the natural log of the likelihood function results in the log-likelihood function as:


$$logL\left(\beta |y_{i}\right)=\overset{n}{\underset{i=1}{\sum }}\left(y_{i}x_{i}^{T}\beta -x_{i}^{T}\beta -log\left(y_{i}!\right)\right)$$ (7)

The model coefficients *β* can be estimated by maximizing the log-likelihood function (Equation 7) using numerical optimization techniques, providing consistent and asymptotically normal estimates of the regression parameters [47].

Recognizing the potential for overdispersion (i.e., fitted variance being larger than the mean), a common occurrence in epidemiological data, we extended our analysis to the Generalized Poisson regression (GPR) model [48]. The GPR model allows for greater flexibility by introducing a dispersion parameter, thereby accommodating the case when the variance exceeds the mean [49]. The key advantage of GPR model is its ability to simultaneously account for overdispersion, underdispersion, and cluster heterogeneity in disease count data from various locations. [50]. The probability mass function (PMF) of the Generalized Poisson (GP) distribution is expressed as [48]:


$$f(Y_{i}=y_{i}|\mu_{i},\alpha )={\left(\frac{\mu_{i}}{1+\alpha \mu_{i}}\right)}^{y_{i}}\frac{{\left(1+\alpha y_{i}\right)}^{y_{i}-1}}{y_{i}!}exp\left(-\frac{\mu_{i}\left(1+\alpha y_{i}\right)}{1+\alpha y_{i}}\right),y_{i}=0,1,\ldots ,$$ (8)

where $Y_{i}$ is the count of Mpox cases, and $\mu_{i}$ is the mean (expected) number of cases in the *i* th PHU, α>0, and 0≤α<1. The mean of the GPR is $E\left(Y_{i}\right)=\mu_{i}=\mu_{i}{\left(1-\alpha \right)}^{-1},$ and the variance is Var $E\left(Y_{i}\right)=\mu_{i}{\left(1-\alpha \right)}^{-3}=\mu_{i}{\left(1-\alpha \right)}^{-2}=\varphi \mu_{i}$. The term $\varphi ={\left(1-\alpha \right)}^{-2}$ is a dispersion factors, and it is utilized in the GP mass function. The parameter *α* serves as a dispersion parameter, and it is evident that when α=0, the GPR model reduces to standard PR model, where the variance equal the mean, when α<0, the GPR model adjusts for underdispersion, where the variance is less than the mean, and when α>0, the GPR model accounts for overdispersion, where the variance exceeds the mean. Other parameterizations of the generalized Poisson distribution exist, but their application is reserved for future research.

The Mpox count cases are related to the predictors $x_{i}$ via the log-link function as:


$$Y_{i}\sim \text{GPoiss}\left(\mu_{i}\right),\text{log}\left(\mu_{i}\right)=\overset{n}{\underset{i=1}{\sum }}x_{ij}\beta_{j}+\alpha$$ (9)

where $x_{ij}$ is the *i* th Mpox count of the *j* th covariate, *n* is the number of covariates in the GPR model, and $\beta_{j}$ is the *j* th regression coefficient. The maximum likelihood estimates of the GPR parameters $\left(\hat{\beta },\hat{\alpha }\right),$ are obtained by maximizing the log-likelihood function $l\left(\beta ,\alpha \right),$ as:


$$L\left(\beta ,\alpha \right)=\overset{n}{\underset{i=1}{\sum }}y_{i}log\left(\frac{\mu_{i}}{1+\alpha \mu_{i}}\right)+\left(y_{i}-1\right)log\left(1+\alpha y_{i}\right)-\frac{\mu_{i}\left(1+\alpha y_{i}\right)}{1+\alpha \mu_{i}}-log\left(\Gamma \left(y_{i}+1\right)\right)$$ (10)

The Newton-Raphson numerical iteration approach is mainly used to maximize the log-likelihood function, where the first and second derivatives of the log-likelihood are required [48]. The sequential iteration procedure is implemented to obtain the maximum likelihood estimates $\left(\hat{\beta },\hat{\alpha }\right).$ Finally, we used SAS version 9.4 version for all the non-spatial analysis. model.

### Test for overdispersion and model fit assessment

Overdispersion occurs when the variance of the response variable exceeds the mean, violating the assumption of the standard PR model. According to [51], the presence of over-dispersion in the PR model can be assessed by conducting a Pearson Chi-square $\left(\chi^{2}\right)$ goodness-of-fit test as:


$$\chi^{2}=\overset{n}{\underset{i=1}{\sum }}\frac{{\left(y_{i}-{\hat{\mu }}_{i}\right)}^{2}}{Var\left({\hat{\mu }}_{i}\right)}$$ (11)

This statistic follows a Chi-square distribution with n−k−1 degrees of freedom, where *k* denotes the number of predictors. The PR model is considered overdispersed if the Pearson Chi-square $\left(\chi^{2}\right)$ value divided by the degrees of freedom exceeds one, accompanied by a significant result (p<0.005).

For model assessment, comparing the Standard PR model and GPR model is essential when overdispersion is detected. This comparison is critical for identifying the most suitable and well-fitted model for the Mpox data, ultimately leading to more robust estimates of the effects of the predictor variables. Model fit was assessed using the Log Likelihood, Akaike Information Criterion (AIC), Akaike Information Criterion corrected (AICc), and Bayesian Information Criterion (BIC), where lower values indicate better model fit [52]. All statistical analyses were conducted using SAS (version 9.4, Cary, NC, USA) [53], and presented as incidence rates of IRRs for Mpox, with corresponding 95% confidence intervals (CIs). Confidence intervals that did not include unity were considered statistically significant.

## Results

### Descriptive results

**Fig 2:**
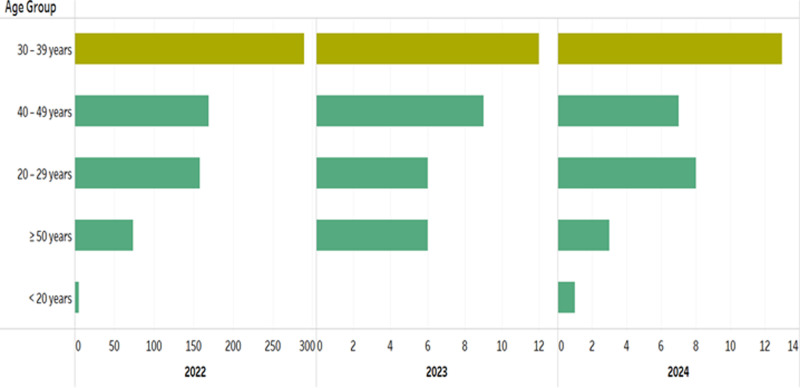
Distribution of Mpox by age group (2022-2024).

**Fig 3:**
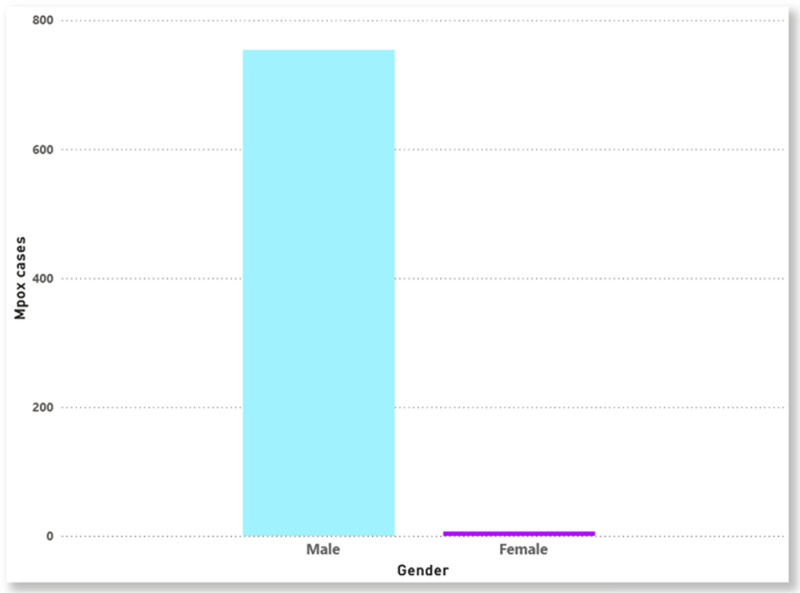
Distribution of Mpox by gender (2022-2024).

**Fig 4:**
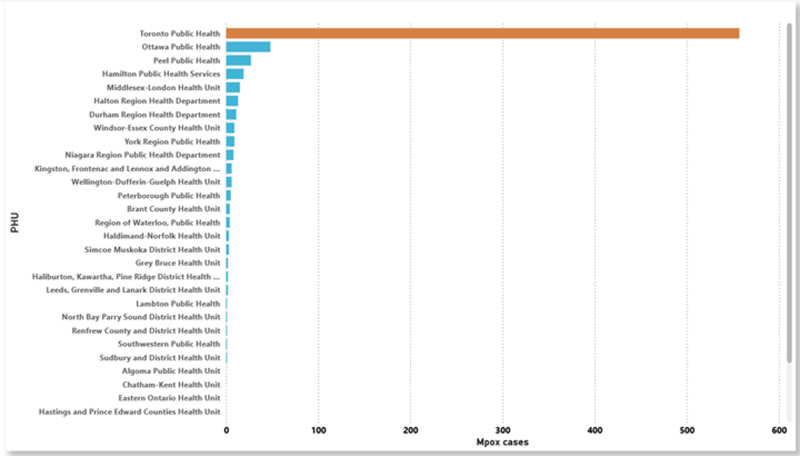
Bar chart of Mpox cases across the Ontario PHUs.

A total of 758 Mpox cases were notified in Ontario from 2022 to March 2024. **Fig 2** and **Fig 3** display the distribution of Mpox cases by age group and gender, respectively. The highest Mpox infection rate is among individuals aged 30–39 years, accounting for ~  67% of Mpox cases (**Fig 2**). This age group had the highest or similarly high rates of Mpox infection across all public health units. The data also indicates that males are more frequently affected by Mpox infections than females (**Fig 3**). As evidenced in **Fig 4**, the bar chart distribution of Mpox incidence rates across Ontario indicated that the highest Mpox incidence rates were recorded in Toronto as compared to other PHUs.

### Spatial analysis results

**Fig 5:**
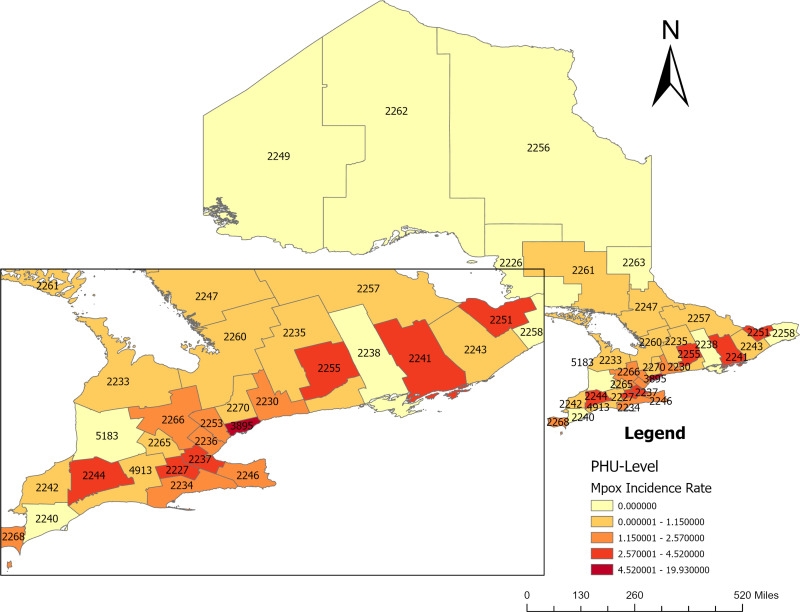
Choropleth map of geographical distribution of Mpox incidence rate in Ontario (Incidence rate: case per 100,000 population) (PHU names are available in Fig 1). Shape file source: Statistics Canada 2021, URL: https://www12.statcan.gc.ca/census-recensement/2021/geo/sip-pis/boundary-limites/index2021-eng.cfm?year=21. Map produced by authors using ArcGIS Pro version 3.0.3.

The Choropleth map in **Fig 5** displays the geographical distribution of the Mpox incidence rate in Ontario at the PHU level. Seven of the 34 PHUs have incidence rates for Mpox greater than 2.6 per 100, 000 population, including Toronto (3895), Ottawa (2251), Peterborough (2255), Hamilton (2237), Kingston (2241), Brant County, and Middlesex-London (2244) PHUs, all of those from Eastern, Central-east, Central-west and South-west health unit regions. Another seven of the remaining 27 PHUs have incidence rates for Mpox greater than 1.2 per 100, 000 population including Wellington-Dufferin-Guelph (2266), Peel (2253), Halton region (2236), Windsor-Essex County (2268) Haldimand-Norfolk (2234), Durham (2230), and Niagara region (2246) PHUs, five of those from Central-west health unit region. Other PHUs had considerably low and no Mpox incidence rate was recorded in the North-west and North-east health unit regions (**Fig 5**).

**Fig 6:**
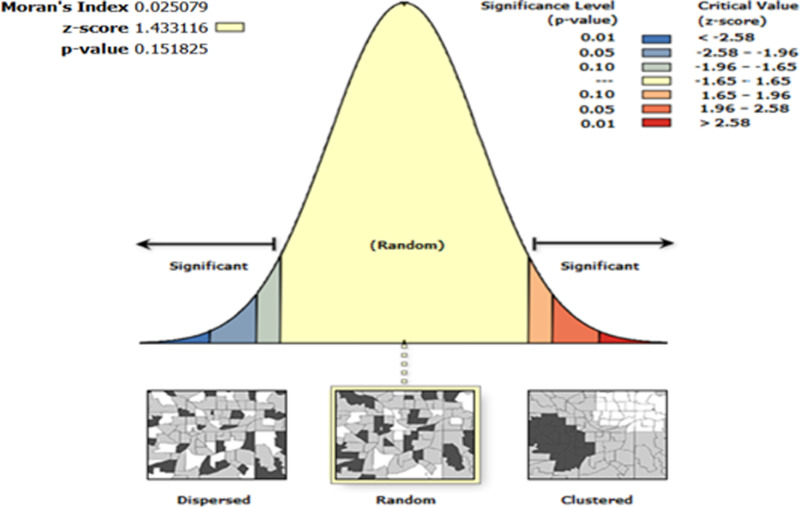
Global spatial autocorrelation analysis of Mpox incidence rate in Ontario (global Moran’s I). Shape file source: Statistics Canada 2021, URL: https://www12.statcan.gc.ca/census-recensement/2021/geo/sip-pis/boundary-limites/index2021-eng.cfm?year=21. Fig produced by authors using ArcGIS Pro version 3.0.3.

The global Moran’s Index value was $\left(Moran^{\prime }sI=0.025079,z-score=1.433116,p-value=0.151825>0.05\right)$ for Mpox incidence rate as shown in **Fig 6**. The result of the global Moran’s I indicated no presence of statistically significant spatial autocorrelation in Mpox incidence rate over the whole study region (Ontario PHUs). Given the z-score of 1.433116, the Mpox spatial pattern does not appear to be significantly different than random, meaning that the Mpox incidence rate in the model is randomly distributed at the PHU level. The specific value is displayed in **Fig 6**. Therefore, applying spatial smoothing in this context would be misleading, as it assumes a spatial correlation does not exist [41].

**Fig 7:**
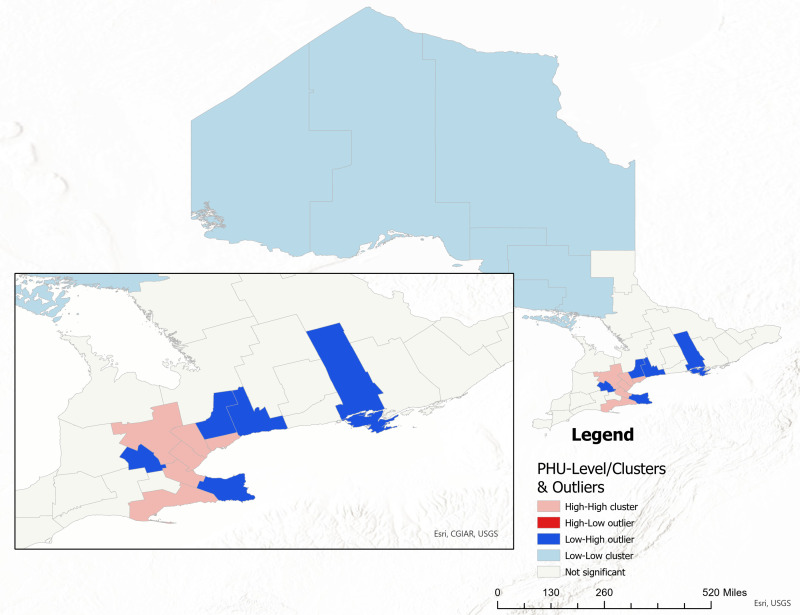
Local spatial autocorrelation analysis of Mpox incidence rate in Ontario (local Moran’s I) (PHU names are available in Fig 1). **Shape file source: Statistics Canada 2021, URL:** https://www12.statcan.gc.ca/census-recensement/2021/geo/sip-pis/boundary-limites/index2021-eng.cfm?year=21. Map produced by authors using ArcGIS Pro version 3.0.3.

The Local Moran’s I statistic indicated that several Public Health Units (PHUs) in Ontario, including Toronto (3895), Peterborough (2255), Kingston (2241), Ottawa (2251), Peel (2253), Wellington-Dufferin-Guelph (2266), Middlesex-London (2244), Windsor-Essex County (2268), Halton Region (2236), Brant County (2227), Hamilton (2237), and Haldimand-Norfolk (2234), exhibited statistically significant High-High clusters for Mpox incidence throughout the study period (Fig 7**).**

**Fig 8:**
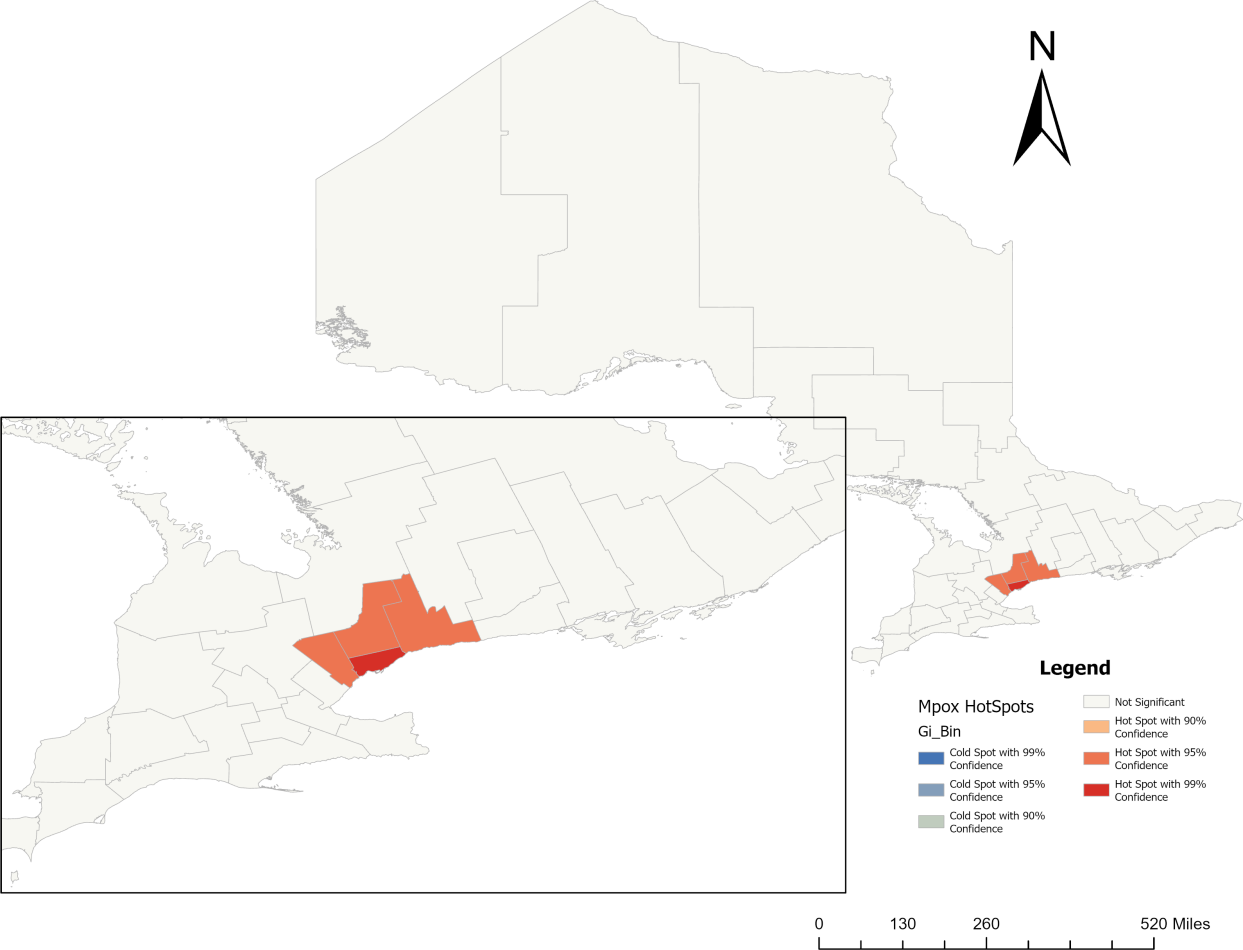
Getis-Ord Gi * hotspot analysis of Mpox incidence rate in Ontario (PHU names are available in Fig 1). **Shape file source: Statistics Canada 2021, URL:** https://www12.statcan.gc.ca/census-recensement/2021/geo/sip-pis/boundary-limites/index2021-eng.cfm?year=21. Map produced by authors using ArcGIS Pro version 3.0.3.

**Fig 8** presents a hotspot analysis for Mpox in Ontario using the local Getis-Ord Gi * statistic. The map clearly shows that Mpox incidence hotspots are primarily located in Toronto, with a 99% confidence level of significance. However, the cluster overlaps with neighboring PHUs, namely Durham Region (2230), Peel (2253), and York Region (2270), which have a statistically significant level of 90% (**Fig 8**). These results suggest that Toronto has neighborhoods with a high incidence of Mpox, potentially due to sociodemographic and marginalized index factors. Therefore, targeted outreach programs focusing on Mpox screening interventions and resource allocation are recommended.

**Fig 9:**
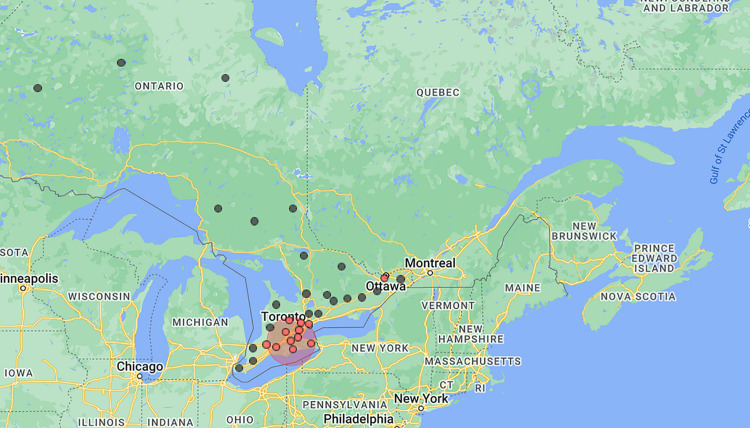
Spatial scan statistic calculated in SaTScan to detect significant Mpox local clusters. **Shape file source: Statistics Canada 2021, URL:** https://www12.statcan.gc.ca/census-recensement/2021/geo/sip-pis/boundary-limites/index2021-eng.cfm?year=21. Map produced by authors using **SaTScan** version 10.1.3.

**Fig 10:**
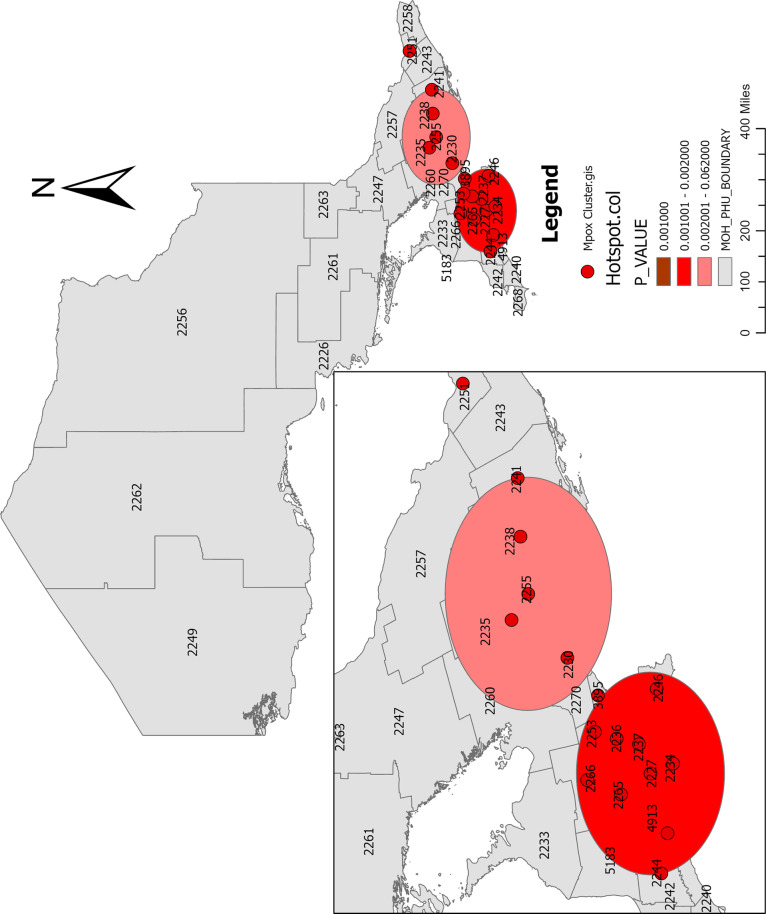
Spatial scan statistic calculated in SaTScan to detect significant Mpox local clusters (visualized in ArcGIS Pro). **Shape file source: Statistics Canada 2021, URL:** https://www12.statcan.gc.ca/census-recensement/2021/geo/sip-pis/boundary-limites/index2021-eng.cfm?year=21. Map produced by authors using ArcGIS Pro version 3.0.3.

The spatial scan statistic revealed two spatial clusters (**Figs 9 and 10**). The primary cluster was located in one PHU (**Toronto**). There were 557 Mpox cases compared to 149 expected cases. Thus, the ratio between the observed and the expected Mpox cases was 3.74. The p-value was 0.0001, smaller than 0.05 significant level, which indicated that the cluster was highly significant. The relative risk (RR) for the population inside the cluster compared to the population outside the cluster was 11.34, indicating that the risk of Mpox within Toronto was higher than locations outside it (neighboring PHUs). The RR is the estimated risk within the cluster divided by the estimated risk outside the cluster. The Log likelihood ratio of the primary cluster was 511.975.

The spatial scan analysis also revealed that two secondary cluster, which is the most likely clusters, were located (a) in one PHU (Ottawa), and (b) overlapped by Toronto and Hamilton PHUS (the overlapped cluster is made up of districts in Kitchener, Brampton, Guelph, Mississauga, Brantford, and London). The first secondary cluster (a) has 48 number of observed Mpox cases was, compared to 17.89 expected Mpox cases. Thus, the ratio between observed and expected cases was 2.68. According to these calculations, the relative risk (RR) inside the cluster was 3.21, while the Log likelihood ratio was 19.884. The p-value was smaller than 0.05, indicating that the cluster was highly significant. The relative risk for the population inside the cluster compared to the population outside the cluster was 3.21, indicating that the risk of Mpox in Ottawa PHU was higher than locations outside the PHU (**Figs 9 and 10**).

For the second secondary cluster (b), which is made up of nine local districts, the number of observed Mpox cases was 100, compared to 73 expected Mpox cases. Thus, the ratio between the observed and expected Mpox cases was 1.37. According to these computations, the RR inside the cluster was 2.07, while the Log likelihood ratio was 9.649. The p-value was 0.0004, smaller than 0.05 level of significance, indicating that the cluster was also significant. The relative risk for the population inside the cluster compared to the population outside the cluster was 2.07, indicating that the risk of Mpox in these areas was higher than locations outside the areas (**Figs 9 and 10**).

### Non-spatial statistical analysis

**Fig 11:**
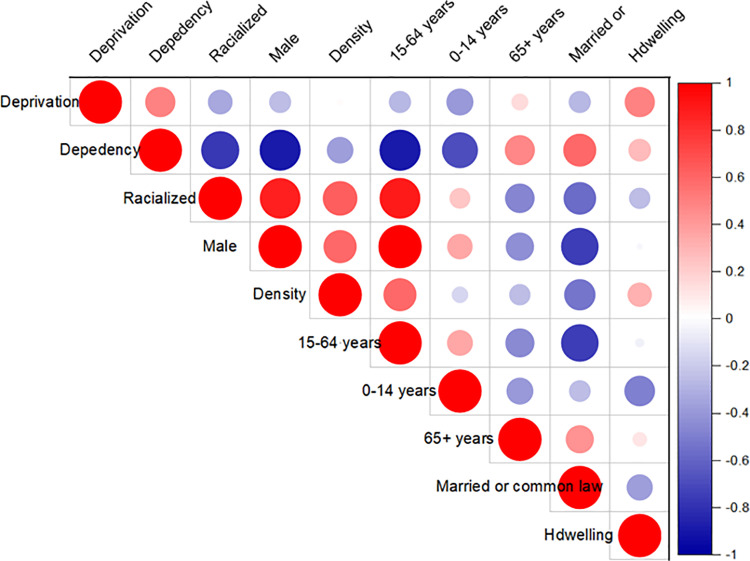
A Pearson correlation matrix illustrates the interdependence of the explanatory variables. Correlation coefficient values may be negative (-1) or positive (+1). If the correlation value is less than zero, it is weak, if it is larger than zero.

The initial Global Moran’s I test for spatial autocorrelation (**Fig 6**) indicated an absence of spatial correlation, suggesting that spatial dependence does not need to be accounted for in the data. Before fitting the Mpox counts to the standard PR model, we conducted an exploratory analysis to examine the multicollinearity among the variables described in **Table 1**, as well as the confounding risk factors using a Pearson’s correlation analysis (**Fig 11**), which might introduce redundancy into the model [54]. We utilized the variance inflation factor (VIF) to assess multicollinearity, which is a problem in regression modelling [54]. The VIF measures how much multicollinearity inflates the variance of regression coefficients. In this analysis, sociodemographic variables with VIF values above 10, indicating high collinearity with ON-Marg factors, were removed from the standard PR model.

**Fig 12:**
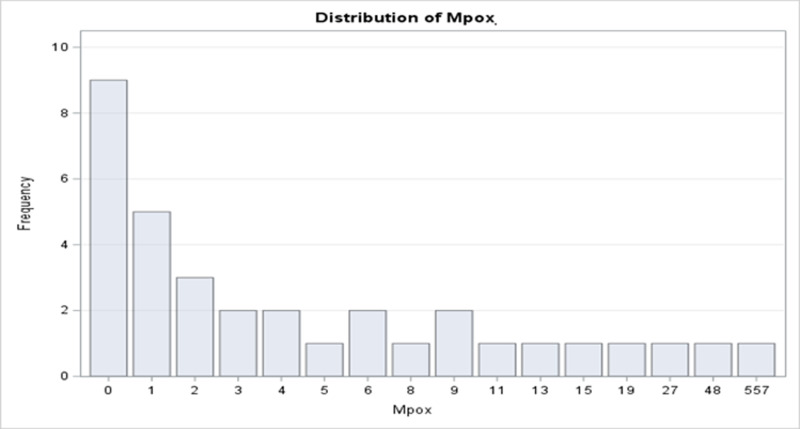
Histogram of distribution of Mpox counts in Ontario PHUs.

The Mpox count data were initially fitted using a standard Poisson regression (PR) model. Overdispersion, which was assessed via $\chi^{2}$ statistic, showed variance exceeding the mean (Dispersion parameter >1) as seen in **Table 2**. **Fig 12** further highlighted excess zeros and a skewed distribution. Results from the standard PR analysis in **Table 3**, indicated significant p− values for all predictors, suggesting inflated estimates due to overdispersion. Therefore, the standard PR model is unsuitable for this data, and alternative models like generalized Poisson regression (GPR) would be more appropriate.

**Table 2:** Goodness-of-fit based on test of overdispersion, Pearson $\chi^{2}$ and deviance.

Model	DF	Pearson $\chi^{2}$	Dispersion	Deviance
PR	29	88.535	3.053	89.535
GPR	29	32.3118	1.114	39.137
*Dispersion= $\frac{\text{Pearson }\chi^{2}}{DF}$				

**Table 3:** Standard Poisson regression coefficients for factors influencing Mpox counts at PHU level in Ontario.

Variables	Parameter estimate	Standard error	IRR	95% CI for IRR	P-value
**Population density**	1.374	0.033	3.951	[3.704, 4.215]	<0.001
**(Racialized and Newcomer Populations or ethnic concentration or percentage of visible minority)**	1.987	0.051	7.293	[6.599, 8.060]	<0.001
**Material resources (deprivation)**	2.247	0.166	9.459	[6.832, 13.097]	<0.001
**Households and Dwellings (Residential Instability)**	2.340	0.129	10.381	[8.062, 13.368]	<0.001
**Increasing age (redundancy)**	-0.758	0.120	0.469	[0.370, 0.5928]	<0.001
**Married or common-law partner**	-0.812	0.018	0.444	[0.429, 0.459]	<0.001

To compare the standard PR and GPR models, Log-likelihood, AIC, AICc, and BIC values were assessed as shown in **Table 4**. The GPR model, with a higher log-likelihood (3268.5252 vs. 1595.0767 for PR), demonstrated a better fit. Additionally, the GPR model had lower AIC (163.6763), AICc (183.5366), and BIC (174.3608) values, confirming its superiority over the PR model. Furthermore, the GPR model’s dispersion parameter (1.114) was much closer to the ideal value of one, compared to the PR model (3.053) in **Table 2**, indicating that the GPR model effectively accounted for overdispersion in the Mpox count data.

**Table 4:** Model comparison (Criteria for assessing goodness- of-fit).

Description	AIC (smaller is better)	AICc (smaller is better)	BIC (smaller is better)	Log Likelihood
**Standard PR**	178.8521	189.1598	201.3582	1595.0767
**GPR**	**163.6763**	**183.5366**	**174.3608**	**3268.5252**

### Results of the generalized Poisson regression (GPR) analysis

Results in **Table 5** provide estimates of the effect of socio-environmental variables for Mpox incidence in Ontario using the GPR model. To address collinearity in the GPR model and enhance the robustness of the findings, the proportion of the racial population (ethnic concentration) was selected as a representative variable for both population density and the percentage of married or common-law partners. Accordingly, among the predictor variables in the final model, proportion of the racialized population (minority or ethnic concentration) $\left(IRR=9.478;95\%CI=5.062-17.747\right)$, male gender $\left(IRR=5.150;95\%CI=1.159-8.324\right)$, and residential instability $\left(IRR=14.112;95\%CI=6.596-30.189\right)$, were statistically significantly and positively associated with increased risk of Mpox incidence in Ontario. According to the results, increasing age and material resources were not significant predictors of Mpox in the GPR model.

**Table 5:** Generalized Poisson Regression coefficients for factors influencing Mpox incidence at PHU level in Ontario.

Variable	Estimate	SE	IRR	95% CI for IRR	p-value
**Intercept**	2.136	0.142	8.466	[6.409, 11.182]	< 0.001***
**(Racialized and Newcomer Populations or ethnic concentration or percentage of visible minority)**	2.249	0.320	9.478	[5.062, 17.747]	< 0.001***
**Material resources (deprivation)**	-1.102	0.657	0.332	[0.091, 1.204]	0.093
**Households and Dwellings (Residential Instability)**	2.647	0.388	14.112	[6.596, 30.189]	<0.001***
**Increasing age (redundancy)**	0.509	0.678	1.664	[0.440, 6.283]	0.453
**Gender (Male)**	1.639	0.245	5.150	[1.159, 8.324]	<0.001***

*p < 0.05,

**p < 0.01,

***p < 0.001, IRR=Relative Risk Ratio, SE=Standard Error, CI=Confidence Interval

### Significant interaction effects

**Fig 13:**
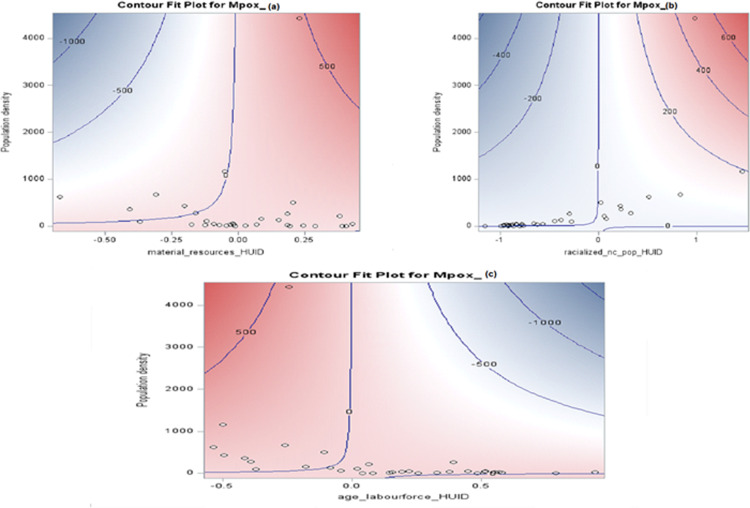
Interaction effect of population density, material deprivation and Mpox (a); population density, ethnic concentration and Mpox (b); population density, age and Mpox (c). The Mpox outcome is plotted as the contours. The blue areas denote lower counts of Mpox, while the redder areas denote higher counts, and the contours are labeled with the corresponding Mpox counts.

PHU-level specific associations between Mpox occurrence and socio-environmental factors were examined modeling an additional interaction term. The interaction effects (**Fig 13**) show a clear increasing trend in the rate of Mpox infection as population increased. It is worth noting that those living in the most ethnically diverse (racial population) have a considerably higher rate of Mpox $\left(p-value<0.001\right)$. The result indicated that increasing population density and proportion of these visible minority groups statistically significantly increased the risk of Mpox in high-risk areas $\left(p-value<0.001\right).$ Interestingly, as age increases the risk of Mpox in the population decreases $\left(p-value<0.001\right)$, and as material deprivation increases in a lower population density, Mpox infection also increases $\left(p-value<0.001\right).$

## Discussion

In this study, we examined the geographical distribution and socio-environmental factors contributing to the high incidence rate of Mpox in Ontario. Our spatial analysis detailed the Mpox patterns at the Public Health Unit (PHU) level from May 1, 2022, to March 31, 2024. The findings revealed significant localized spatial heterogeneity in Mpox distribution, indicating that local factors play a critical role in influencing the incidence of Mpox in specific PHUs. However, the Global Moran’s I statistic indicated a random spatial distribution of Mpox cases on a broader scale, suggesting that cases are not clustered globally. This lack of global clustering may reflect diverse epidemiological dynamics and varying public health responses across different PHUs.

Our analysis identified Toronto as the primary Mpox hotspot, a region characterized by a significant migrant population and ethnic concentration. With a population of approximately 2.8 million and various socioeconomic factors at play [25], Toronto emerged as a key area of concern. The application of spatial correlation methods proved effective in pinpointing priority Public Health Units (PHUs) for targeted Mpox vaccination strategies, interventions, and resource allocation in Ontario. Furthermore, our findings suggest that socio-environmental factors and marginalization levels within PHUs are linked to Mpox incidence, with higher infection risks observed in areas with greater population density and marginalization, consistent with comparable studies in the United States and Brazil that found higher rates of Mpox infection in densely populated areas [3,4,6].

The incidence rate of Mpox in Ontario was statistically significantly associated with a higher proportion of ethnic concentration (racialized groups, migrants, or visible minorities), increased residential instability, and the male gender. These findings align with existing knowledge about ecological and location-specific risks for Mpox, providing evidence that high-risk areas at the Public Health Unit (PHU) level, along with localized neighborhood structures and socio-environmental characteristics, are important determinants of Mpox infection risk [1,2,6].

The associations identified in this study between human Mpox infection and PHU-level measures of marginalization provide the first evidence of socio-environmental risk factors for Mpox in Ontario, Canada. The ON-Marg factors, such as ethnic concentration and residential instability defined in **Table 1**, reflect complex sociodemographic dimensions that complicate the assessment of individual contributions to Mpox risk. Notably, residential instability was associated with increased Mpox incidence, as unstable housing can lead to decreased access to healthcare, increased stress, and higher rates of transmission in densely populated areas. Additionally, a higher proportion of ethnic concentration or visible minority groups was statistically significantly linked to increased Mpox risk. These findings may be explained by population density, which enhances human-environment interactions in urban areas. The findings align with recent studies indicating that regions with dense populations and high migration rates face greater Mpox infection risk [4,55–58].

Findings from COVID-19 studies in Ontario, where elevated cases were linked to crowded living conditions, low-income areas, and high-contact jobs among immigrant and socially deprived groups [59–64], suggest similar risk factors could apply to Mpox. These social determinants contribute to health inequities, increasing vulnerability to Mpox in these populations.

The result of this study collaborates previous studies on Mpox, suggesting that younger and middle-aged adults were more vulnerable to Mpox infection, especially with male gender [22,62]. Therefore, controlling population activities and monitoring public health management and vaccination in such areas must target younger age groups especially the male gender. At the same time, it is necessary to improve the public’s awareness of the Mpox virus, understanding the changing epidemiology of the disease is also the key to formulating prevention and control strategies. Minimizing human interaction in areas with frequent suspicious host activities can reduce the likelihood of introducing Mpox into human populations; if Mpox is not repeatedly introduced, the infection will eventually cease in Toronto and other high-risk areas.

After adjusting for other variables in the GPR model, material deprivation and increasing age were identified as negative predictors of Mpox incidence in Ontario, although these associations were not statistically significant. This suggests that higher Mpox incidence may occur in PHUs with better socioeconomic indicators, as household instability was a significant risk factor. Additionally, increasing age, reflected in the proportion of the population aged 65 and older and the dependency ratio, may indicate that older demographics in these areas are less susceptible to Mpox, potentially due to lower rates of interpersonal contact.

Access to healthcare and existing inequities add complexity to our understanding of Mpox infection risks. Research indicates that individual income levels alone do not have a significant impact on the likelihood of contracting Mpox; instead, the geographic location of a person’s residence is a critical factor [64,65]. This suggests that where individuals reside, particularly in relation to healthcare resources and community support, can greatly influence their vulnerability to Mpox infection, highlighting the importance of addressing these spatial and systemic inequities in public health strategies.

## Study’s strength and limitation

There are several limitations to our study. First, we used a limited number of variables based on publicly available data at the PHU level. While Ontario marginalized index used various census variables for 2021 to define dimensions of marginalization, some details are not well captured like the issue of racism, and health literacy. Similarly, given that ON-Marg index factors only explain a portion of Mpox incidence, it is important to consider other environmental, behavioral, and biological factors that impact a population’s risk of contracting Mpox. Furthermore, this study was not structured to establish causal relationships, nor does it consider all the factors that might contribute to a causal pathway. However, our study is first to use a comprehensive marginalization index as socio-environmental factors to better understand the incidence of Mpox in Ontario. Further research is needed to clarify our findings and their public health implications by incorporating additional variables not included in this study.

## Conclusion

This study has demonstrated geographical distribution and the impact of marginalization on Mpox incidence across the PHUs in Ontario. Our study showed that most Mpox incidences in Ontario occurred in PHUs with better socioeconomic indicators and higher population density (higher proportion racial and ethnic minority groups). Additionally, household dwelling (residential instability) was found to be associated with increased risk of Mpox. Measures to enhance Mpox diagnosis and promote health equity among socioeconomically vulnerable populations, including individuals from racial and ethnic minority backgrounds, need to be put into action. This study offers valuable insights for resource allocation and policymaking for Mpox control and prevention. Further studies and policy intervention will be invaluable not only to identify and support socially marginalized populations in Ontario but also to address possible racial issues on a large scale in context of Mpox control and resource allocation.
